# Optimal virtual water flows for improved food security in water-scarce countries

**DOI:** 10.1038/s41598-021-00500-6

**Published:** 2021-10-25

**Authors:** Saman Maroufpoor, Omid Bozorg-Haddad, Eisa Maroufpoor, P. Winnie Gerbens-Leenes, Hugo A. Loáiciga, Dragan Savic, Vijay P. Singh

**Affiliations:** 1 Faculty of Agricultural Engineering and Technology, Department of Irrigation and Reclamation, College of Agriculture and Natural Resources, University of Tehran, Karaj, Tehran, Iran; 2 Department of Water Engineering, Faculty of Agriculture, University of Kurdistan, Sanandaj, Iran; 3 Integrated Research on Energy, Environment and Society (IREES), University of Groningen, 9747 AG Groningen, The Netherlands; 4 Department of Geography, University of California, Santa Barbara, CA 93016-4060 USA; 5 KWR Water Research Institute, Nieuwegein, The Netherlands; 6 Centre for Water Systems, University of Exeter, Exeter, UK; 7 Faculty of Engineering and Built Environment, Universiti Kebangsaan Malaysia, Bangi, Malaysia; 8 Caroline & William N. Lehrer Distinguished Chair in Water Engineering, Department of Biological and Agricultural Engineering and Zachry Department of Civil and Environmental Engineering, Texas A&M University, 321 Scoates Hall, 2117 TAMU, College Station, TX 77843-2117 USA

**Keywords:** Climate sciences, Ecology, Environmental sciences, Environmental social sciences, Hydrology, Natural hazards, Engineering, Mathematics and computing

## Abstract

The worsening water scarcity has imposed a significant stress on food production in many parts of the world. This stress becomes more critical when countries seek self-sufficiency. A literature review shows that food self-sufficiency has not been assessed as the main factor in determining the optimal cultivation patterns. However, food self-sufficiency is one of the main policies of these countries and requires the most attention and concentration. Previous works have focused on the virtual water trade to meet regional food demand and to calculate trade flows. The potential of the trade network can be exploited to improve the cropping pattern to ensure food and water security. To this end, and based on the research gaps mentioned, this study develops a method to link intra-country trade networks, food security, and total water footprints (WFs) to improve food security. The method is applied in Iran, a water-scarce country. The study shows that 781 × 10^6^ m^3^ of water could be saved by creating a trade network. Results of the balanced trade network are input to a multi-objective optimization model to improve cropping patterns based on the objectives of achieving food security and preventing water crises. The method provides 400 management scenarios to improve cropping patterns considering 51 main crops in Iran. Results show a range of improvements in food security (19–45%) and a decrease in WFs (2–3%). The selected scenario for Iran would reduce the blue water footprint by 1207 × 10^6^ m^3^, and reduce the cropland area by 19 × 10^3^ ha. This methodology allows decision makers to develop policies that achieve food security under limited water resources in arid and semi-arid regions.

## Introduction

### Research background

Global food security is a prerequisite for sustainable development^1,2^. The FAO^3^ projected an annual growth of agricultural demand by 1.1% for the period 2005–2050. Population growth, rising per capita consumption, and changing diets have led to a larger demand for agricultural products and rising crop production^4^. This can be achieved by expanding croplands, improving crop yields, and double cropping^5^. Increasing production is mainly achieved by improving crop yields on a global scale, however, in developing countries (e.g., Iran) cropland expansion will also play an important role in increasing production^6,7^.

Freshwater is essential for agriculture. Expanding crop production implies the increase in the use of water, land, fertilizers, pesticides, energy, human labor, and financial investments^8^. Often, water is a limiting factor for crop growth as is demonstrated by the dominant position of agriculture in global freshwater use. Water productivity and consequent water scarcity are key factors that may threaten sustainable social and environmental development, and, ultimately food security in arid areas^9,10^. Agricultural production around the world increased by a factor of 2.5–3 between 1960 and 2010, which was a remarkable achievement^8^. At the same time, irrigation accounts for 70% of all freshwater withdrawals^8^. It is noteworthy that 92% of Iran's water resources are used in the agricultural sector.

Rising global food production has put pressure on freshwater resources, especially in countries with high growth rates of food demand^3^. For example, in the Middle East and North Africa (MENA) regions, where biophysical conditions are less suitable for agriculture than in regions with sufficient rainfall, food security is threatened by increasing population and consumption^11^.

Freshwater applied to agriculture comes from precipitation (often termed green water), or from rivers, lakes, or groundwater—often termed blue water^12^. Water shortages in many countries occur during part of the year, and in some countries during the entire year^13^. One way to supply irrigation water is to exploit groundwater resources. However, caution is needed because groundwater is overexploited in many regions^14–16^. Furthermore, water stress, which creates pressure on the quantity and quality of water resources, occurs when demand exceeds the available amount of water. Water stress can be expressed as the proportion of available freshwater resources that is withdrawn for various uses, or 100 × (Total freshwater withdrawal)/((Total renewable water resources)-(Environmental Flow Requirements))^17^. Iran is a country located in a water-scarce region with water stress reaching 81%, which compares with Saudi Arabia’s water stress of 883% and Spain’s 43%. The average water stress level in Iran is high, yet, there are large differences among its regions. Karandish and Hoekstra^18^, for example, identified five climatic regions with different water availabilities, i.e., humid, dry sub-humid, semi-arid, arid, and hyper-arid region, and showed that the irrigation water requirement, expressed as the blue water footprint (WF) of a crop, depends on the climatic regime where crops are grown. According to the Food and Agriculture Organization Food Summit definition, food security will be achieved when all people have physical and economic access to sufficient, healthy and nutritious food to meet their nutritional needs for a healthy life^19^. The FAO^20^ defined food self-sufficiency as the capacity of country to satisfy its food needs from its own domestic production. Iran is a large agricultural country in the MENA region that has pursued a food self-sufficiency program since 1990^21^. However, this program is based on cropland expansion with little attention to crop water productivity and trade^18^. Therefore, the increase in production in Iran has been achieved based on the maximum use of freshwater resources^22^. Moreover, according to the Iranian Ministry of Agriculture, most of the internationally exported crop types (i.e., wheat, barley, rice, tea, and potato) are also imported in the same year^23^. In addition, regardless of national crop demands and regional potential, regional cropping patterns are mainly based on traditional agricultural methods^24^. Iranian government policies have led farmers to focus solely on crop management but not on water conservation. Production of crops for export is a priority for farmers. However, water resources as a fundamental natural resource for agriculture should not be affected by market allocation^25–27^. Iran must create a trade network that places the agricultural system in line with the country's food demand to achieve food security and self-sufficiency. One approach to fulfil these objectives is to allocate water resources on a regional scale to improve water productivity considering the "Virtual Water Trade" (VWT)^28^. Water consumed in food production is generally considered virtual water (VW)^29^, also referred to as water footprint (WF)^12^. This virtual water is transferred through crops or other products from one place to another (in the form of commercial exchanges), and various exchanges are defined as virtual water flows^30^. The WF concept emphasizes that water has local and global dimensions.

### Literature review and research gaps

Many applications of WFs and trade have considered food security and water^31–34^, and have revealed the feedbacks between water, food, and trade affect socio-economic and environmental conditions^35^. The main focus of WF studies in Iran has been on strategic crops on a regional scale^36–41^. A brief summary of pertinent works follows.

Faramarzi et al.^40^ developed a framework for domestic trade in Iran to achieve an optimal cereal cropping pattern. The main goal was self-sufficiency in wheat production, which based on several scenarios 31–100% of wheat shortage in Iran was provided. Karandish et al.^42^ selected Sistan and Baluchestan as one of the arid provinces of Iran and determined the optimal cropping pattern of 44 crops based on the minimum virtual water. Trade within the province and with regions outside need to meet needs was also assessed. Qasemipour and Abbasi^41^ measured the virtual water content of 37 crops and five livestock products in South Khorasan province. They then used a mass balance equation to estimate the virtual water flow within the province. The results showed that the current agricultural system has caused 206% of water scarcity in South Khorasan province. Ramezani Etedali et al.^37^ studied the optimal cropping pattern of main cereals (wheat, barley, and corn) in 15 provinces of Iran with linear programming. Their goal was to obtain an optimal cropping pattern based on the minimum virtual water of crops.

Date palm is one of Iran's main crops whose footprint index and economic value was evaluated by Bazrafshan et al.^39^. Another important crop in Iran is saffron, whose water footprint in the provinces of Iran was investigated by Bazrafshan et al.^38^. The most suitable provinces for saffron cultivation were determined in terms of the share of water footprint and the amount of virtual water exports abroad.

Ye et al.^33^ developed a multi-objective optimization model to optimize water allocation (including surface water, groundwater, transferred water and reclaimed water) and virtual water resources to several consumption sectors in Beijing, China. The virtual water flows were associated with the trade of five main crops (barley, corn, rice, soy and wheat) and three livestock products (beef, pork and poultry). Their results showed that the ratio of agricultural consumption in total water demand is reduced to 45% by considering the virtual trade of eight agricultural products in water allocation. The imbalance between water supply and demand in this study was eliminated by importing virtual water to water-scarce areas.

Lamastra et al.^43^ investigated bilateral virtual water flows connected to the top ten agri-food products between Italy and China. Comparing the virtual water flow of the top 10 food products showed that the virtual water flows from Italy to China were greater than the water flow in the opposite direction. Recently, Delpasand et al.^44^ reported a virtual water trade management study to maximize economic revenue and reduce the consumption of virtual water in Iran. The latter authors considered the external trade of 6 agricultural crops, which are only a part of Iran's agricultural crops, and 5 industrial products.

A review of the literature reveals that most on virtual water and food security is single-objective and integrates water resources into the optimal cropping pattern. Objective functions generally include economic functions and minimizing the virtual water consumption of products. Among them, food security and self-sufficiency are seen as feedbacks in an optimal cropping pattern. Yet, food security and self-sufficiency have not been used as an objective function or as independent indicators. .

The virtual water trade has been previously used as a tool to compensate for water scarcity. This approach encourages scarce-water regions to import virtual water. In other words, after determining the optimal cropping pattern of regions, virtual water trade is evaluated to meet the demand of regions and to calculate the flow of domestic/international trade for each region. There are no previous works that have used the trade network to modify the cropping pattern to meet the objective of food and water security. Likewise, previous works have not addressed the modification of export crops’ cultivation in the analysis of food and water security. This works considers the trade network in the analysis of food and security. This constitutes this study’s innovation.

### Study objectives

The present study develops a trade network in Iran to support self-sufficiency and avoid exporting crops that have to be imported again in the same year, avoiding unnecessary trade-flows. The results of the trade network are used for the optimization model. The method optimizes crop production taking spatial features and water availability into account and including 31 Iranian provinces and 51 crops. The crop optimization method relies on a multi-objective optimization approach [termed the Non-dominated Sorting Genetic Algorithm-II (NSGA-II)] and achieves the two main objectives: (i) balancing the Iranian agricultural system using water resources efficiently, and (ii) promoting food security and self-sufficiency. The proposed trade network improves the national agricultural system and prevents exporting and importing the same types of crops. It also balances virtual water flows between provinces based on the shortest distance between them.

The output of the optimized trade network is the amount of deficit or surplus per crop (applied to international agricultural exports and imports). This output is input to the optimization model. In the next step the optimization model modifies the cultivation of export crops based on the country's crop deficit. The optimization of export crop cultivation is compatible with the objectives of achieving food security and preventing water crisis, which conforms with macroeconomic policies. Improving the cropping patterns of export crops maximizes the productivity of agricultural water.

## Concepts and methods

### Crop production, water productivity, and virtual water

A method to calculate the water needed for crops is the water footprint (WF). The WF has a color-based classification: green water (precipitation), blue water (ground and surface water), and grey water (water to dilute polluted water to accepted water quality standards). A manual on how to calculate WFs has been published^12^. Calculations of WFs integrate green and blue crop water use (evapotranspiration by crops) over the growing period of specific crops and express results per unit of yield (m^3^ kg^−1^). The difference between crop-water use and effective rainfall is applied as a proxy for blue WFs when no data on actual irrigation water supply are available. WFs of specific crops vary greatly among countries, and even within countries^45^. This means that water can be saved when crops are smartly traded. This may also be possible within a country if crops are grown where water productivity is the highest.

### Calculation of the water footprint

Water footprints (WFs) are calculated as green and blue water footprints (*WF*_*green*_, *WF*_*blue*_, respectively) adopting the method from the WF manual^12^. This study assumes that the difference between crop water requirement and evapotranspiration of green water (ET_Green_) in crops is equal to the evapotranspiration of blue water (ET_blue_); therefore, crop water requirements are met with irrigation water. The crop water requirements are estimated with the Food and Agriculture Organization's CROPWAT model^46^. The selected methods for calculating the reference evapotranspiration (*ET*_0_) and effective precipitation (*P*_*eff*_) are the FAO Penman–Monteith method^47,48^ and the USDA’s SCS method^48^, respectively. Calculations were performed at the provincial scale for each crop. Equations (1) through (4) are applied to calculate *WF*_*green*_ and *WF*_*blue*_ for the crops included in this study:

Actual crop evapotranspiration from reference evapotranspiration:

1 $$ ET_{c} = \sum_{t} {ET_{0} \times K_{c} } $$

Reference evapotranspiration:

2 $$ ET_{0} = \frac{{0.408\Delta \left( {R_{n} - G} \right) + \gamma \frac{900}{{T + 273}}U_{2} \left( {e_{a} - e_{d} } \right)}}{{\Delta + \gamma \left( {1 + 0.34U_{2} } \right)}} $$

3 $$ WF_{green} = 10 \times \frac{{\min \left[ {ET_{c} ,P_{eff} } \right]}}{Y} $$

4 $$ WF_{blue} = 10 \times \frac{{\max \left[ {0,ET_{c} - P_{eff} } \right]}}{Y} $$

where ETc denotes the actual crop evapotranspiration (mm) during the growth period (*t*), *ET*_0_ represents the reference evapotranspiration (mm day^−1^), and *K*_*c*_ denotes the crop coefficient based on crop type and development stages (initial, middle, and late stages). In Eq. (2) *e*_*a*_ (kPa), *e*_*d*_ (kPa), *Δ* (kPa °C^−1^), *G* (MJ m^−2^ day^−1^), *T* (°C), *R*_*n*_ (MJ m^−2^ day^−1^), *U*_2_ (m s^−1^), and *γ* (kPa °C^−1^) denote the saturation vapor pressure, the actual vapor pressure, the slope of the saturation-vapor pressure curve, the soil heat flux, the average air temperature, the net radiation on the crop surface, the wind speed measured at a height of 2 m above ground level, and the psychrometric constant, respectively. Equations (3) and (4) calculate the green and blue water footprints (m^3^ ton^−1^), in which *P*_*eff*_ (mm), *Y* (ton ha^−1^), and 10, are represent effective precipitation, the crop yield, and a conversion factor from mm to m^3^ ha^−1^, respectively. *WF*_*green*_ and *WF*_*blue*_ occur in irrigated cultivation; however, there is only *WF*_*green*_ in rainfed cultivation.

### Optimization of crop production

All the steps of the methods used in this work were coded in MATLAB for use by decision-makers, planners, and interested organizations.

#### Balancing the agricultural system

An internal trade network was created to organize and remedy the weaknesses of the trade network. The lack of a comprehensive trade network has caused the crops to be exported regardless of the country's demands, which eventually leads to the import of the same crops. The production and demand amounts of each crop in each province and their *WF*_*green*_ and *WF*_*blue*_ are determined with the following equations applied to *N* = 51 crops in *J* = 31 provinces:

5 $$ {CP}_{(i,j)} = {ICP}_{(i,j)} + {RCP}_{(i,j)} $$

6 $$ {ICP}_{(i,j)} = \left( {{BCY}_{(i,j)} \times {ICA}_{(i,j)} } \right) $$

7 $$ {RCP}_{(i,j)} = \left( {{GCY}_{(i,j)} \times {RCA}_{(i,j)} } \right) $$

8 $$ {CD}_{(i,j)} = \left( {{PCD}_{i} \times {POP}_{J} } \right) $$

9 $$ {TWF}_{blue(i,j)} = {ICP}_{(i,j)} \times {WF}_{blue(i,j)} $$

10 $$ {TWF}_{green(i,j)} = {ICP}_{(i,j)} \times {WF}_{green(i,j)} $$

where $i=1, 2,\ldots , N;j=1, 2, \ldots, J,$ *CP*_*(i,j)*_ (ton), *ICP*_*(i,j)*_ (ton), *RCP*_*(i,j)*_ (ton), *BCY*_*(i,j)*_ (ton.ha^−1^), *GCY*_*(i,j)*_ (ton.ha^−1^), *ICA*_*(i,j)*_ (ha), *RCA*_*(i,j)*_ (ha), *CD*_*(i,j)*_ (ton), *PCD*_*i*_ (ton.person^−1^), *POP*_*j*_ (person), *TWF*_*blue(i,j)*_ (m^3^), and *TWF*_*green(i,j)*_ (m^3^) denote the production of crop *i* in province *j*, crop production of irrigated land, crop production in rainfed cultivation, irrigated crop yield, rainfed crop yield, irrigated acreage, rainfed areas acreage, demand for crop *i* in province *j*, per capita diet, population of province *j*, the blue WF of crop *i* in province *j* corresponding to irrigated cultivation, and the green WFs of crop *i* in province *j* corresponding to irrigated cultivation, respectively.

*TWF*_*blue(i,j*)_ equals zero in rainfed cultivation, and *TWF*_*green(i,j)*_ is calculated with Eq. (10) based on *RCP*_*(i,j)*_. The deficit or surplus over the demand of the provinces were determined by comparing *CP*_*(i,j)*_ and *CD*_*(i,j)*_ for each crop in each province. Equation (11) implies that *CS*_*(i,j)*_ is the amount of crop *i* supplied in province *j* (ton), which involves the export and import of crops:

11 $$ {CS}_{(i,j)} = {CP}_{(i,j)} - {CD}_{(i,j)} $$

where $i=1, 2,\ldots , N;j=1, 2, \ldots, J$ .The internal trade network is formed once the deficit and surplus for each crop in the provinces is determined, and crops are traded based on the shortest distance between the provinces. The developed trade network would improve the country's agricultural system and reduce transportation costs between the provinces. Each province adds to or subtracts *T*_*i,j*_ (ton) from its crop amounts, where imports imply an addition and exports a subtraction of crop amounts. The internal exports and imports of WFs and the net water footprints trade (*NWFT*) in each province are calculated as follows:

12 $$ {WFT}_{(x,r,i)} = T_{(x,r,i)} \times \left( {{WF}_{green} + {WF}_{blue} } \right)_{(x,i)} $$

13 $$EW{F}_{(x)}={\sum }_{r,i}WF{T}_{(x,r,i)}$$

14 $$ IWF_{(r)} = \sum\limits_{x,i} {WFT_{(x,r,i)} } $$

15 $$ {NWFT}_{(j)} = IWF_{(j)} - EWF_{(j)} $$

where $i=1, 2,\ldots , N;j, x=1, 2, \ldots, J, r=x-1$, *WFT*_*(x,r,i)*_ (m^3^), *T*_*(x,r,i)*_ (ton), *(WF*_*green*_ + *WF*_*blue*_*)*_*(x,i)*_ (m^3^ ton^−1^), *EWF*_*(x)*_ (m^3^), *IWF*_*(r)*_ (m^3^), and *NWFT*_*(j)*_ (m^3^) denote the WFs traded for crop *i* from exporting province *x* to importing province *r*, the amount of crop *i* exported from province *x* to province *r*, the blue and green WFs related to crop *i* in exporting province *x*, the WFs exported from province *x* by the trade of crops, the WFs imported into province *r* by the trade of crops, and the net water footprints trade in province *j*, respectively.

The positive and negative values ​​of *NWFT*_*(j)*_ represent the import and export of WFs to province *j*, respectively. The calculation of the internal trade between provinces with Eq. (11) permits determining the deficits and surpluses for each crop in the provinces nationally. At this juncture the provinces may resort to international trade to cope with deficits and surpluses. However, from this work’s premise of improving food security and self-sufficiency the cropping patterns of surplus crops in the provinces are modified as described in the next section.

#### Modifying exports to optimize the cropping pattern

The multi-objective optimization approach to increase food security and self-sufficiency redirects the resources to be used to cultivate export crops to the cultivation of crops that are in deficit (i.e., whose production is less than demand). This modification of cropping patterns in the provinces is based on their traditional cropping patterns. For this purpose, the internal trade network is linked to the optimization method to manage cropping patterns of the regions based on the output of the trade network, and on the goals of achieving food security and preventing water crisis. These two goals are pertinent in many countries where water scarcity is a limiting factor to achieve food security^49^. Therefore, concerning available agricultural water it is imperative to pay attention to the type of water (green or blue) used. Specifically, *WF*_*blue*_ can be used in several areas of consumption; however, *WF*_*green*_ is not controllable in the same manner. The usage of *WF*_*green*_ by crops depends on the growing season, and the maximum use can be achieved by choosing the optimal crops. Therefore, this work treats *WF*_*green*_ and *WF*_*blue*_ as indicators of water crisis and food security, which were chosen as objective functions. In other words, controlling and managing WFs prevent its waste (thus reducing the water deficit and crisis). Selecting optimal crops based on WFs will increase production and food security. The water crisis and food security serve as the benchmark for comparison between the reference situation (without optimization) and the results of this new method. The reference situation refers to the initial state of food security and water crisis, which occurs before optimizing the cropping patterns.

The food-security objective function is expressed as follows:

16 $$F{S}_{i}=\frac{{\sum }_{j=1}^{J}C{P}_{(i,j)}}{{\sum }_{j=1}^{J}C{D}_{(i,j)}}$$

The water-crisis objective function is written as follows:

17 $${WC}_{j}=\frac{\sum_{i=1}^{N}{TWF}_{blue(i,j)}}{{RWR}_{j}}$$

where $i=1, 2,\ldots, N=51;j=1, 2, \ldots, J=31,$ *FS*_*i*_, *CP*_*(i,j)*_ (ton), *CD*_*(i,j)*_ (ton), *WC*_*j*_, *TWF*_*blue(i,j)*_ (m^3^), and *RWR*_*j*_ (m^3^) denote the food security for crop *i*, production of crop *i* in province *j*, the demand of crop *i* in province *j*, the water crisis in province *j*, the blue WFs of crop *i* in province *j*, and the renewable water resources in province *j*, respectively.

Maximizing the *FS* index and minimizing the *WC* index represent the ideal situation. The maximizing function was converted to a minimization function for the purpose of multiobjective optimization. The final form of the objective functions i given by the following equations:

18 $$Min({Z}_{1})=\frac{1}{N}{\sum }_{i=1}^{N}(1-F{S}_{i})\begin{array}{cc},& where\,\, F{S}_{i}\end{array}=Min\left(\frac{{\sum }_{j=1}^{J}C{P}_{(i,j)}}{{\sum }_{j=1}^{J}C{D}_{(i,j)}},1\right)$$

19 $$Min({Z}_{2})=\frac{1}{J}{\sum }_{j=1}^{J}W{C}_{j}$$

where $i=1, 2,\ldots , N=51;j=1, 2, \ldots, J=31.$ The objective function *Z*_1_ is calculated based on the food security index expressed as an average for all crops, and the objective function *Z*_2_ is calculated as the average of the water crisis indexes in the 31 provinces. Both objective functions are affected by cropping patterns and cultivation areas. The water and land used must be calculated prior to modifying the cropping patterns. The amounts of surplus crops in the provinces and their equivalent water and land are calculated using the following equations:

20 $$ {SCP}_{(i,j)} = Max({CP}_{(i,j)} - {CD}_{(i,j)} + T_{(i,j)} ,0) $$

21 $$ {BCY}_{(i,j)} \times (X_{1(i,j)} \times ICA_{(i,j)} ) + {GCY}_{(i,j)} \times (X_{2(i,j)} \times RCA_{(i,j)} ) = {SCP}_{(i,j)} $$

where $i=1, 2,\ldots, N;j=1, 2, \ldots, J,$ *SCP*_*(i,j)*_, $X_{1(i,j)}$, $X_{2(i,j)}$ denote the surplus crop *i* in province *j* (ton) determined based on demand and trade in the province, and the percentage of crop *i* in province *j* that must be removed from irrigated and rainfed cultivation, respectively. The amount of water and land available for new cultivation are calculated as follows:

22 $$ ICA_{j}^{free} = \sum\limits_{i = 1}^{51} {X_{1(i,j)} \times ICA_{(i,j)} } $$

23 $$ RCA_{j}^{free} = \sum\limits_{i = 1}^{51} {X_{2(i,j)} \times RCA_{(i,j)} } $$

24 $$ TWF_{blue,j}^{free} = \sum\limits_{i = 1}^{51} {{WF}_{blue(i,j)} \times BCY_{(i,j)} } \times ICA_{j}^{free} $$

where $i=1, 2,\ldots , N;j=1, 2, \ldots, J,$ *ICA*_*j*_^*free*^ and *RCA*_*j*_^*free*^ denote the total available area of ​​irrigated and rainfed cultivation (ha) in province *j*, respectively, and *TWF*_*blue,j*_^*free*^ represents the total amount of blue WFs available in province *j* (m^3^). It is noteworthy that the water and land available in irrigated cultivation can be altered. On the other hand, only the available land is controllable under rainfed cultivation.

The objective functions of the proposed method [Eqs. (18) and (19)] were subjected to a set of constraints introduced next.

(i) Modification of the cropping patterns

The available land in each province is allocated to crops that feature a deficit in the country and are part of the traditional cropping patterns of the provinces. The set of cultivable crops is determined using the following equation:

25 $$ P = \left\{ {p\left| {p \in i,\sum_{j = 1}^{31} {SCP_{(p,j)} < 0} } \right.} \right\} $$

where *p* denotes the set of crops with deficit conditions in the country and *SCP*_*(i,j)*_ was defined above. Letting traditional irrigated and rainfed cropping patterns be denoted by *A*_*j*_ and *B*_*j*_ in province *j*, respectively, the set of irrigated and rainfed crops cultivable in province *j* was calculated as follows:

26 $$ IC_{j} = P \cap A_{j} \begin{array}{*{20}c} {} & {(j = 1,2,3,\ldots,31)} \\ \end{array} $$

27 $$ RC_{j} = P \cap B_{j} \begin{array}{*{20}c} {} & {(j = 1,2,3,\ldots,31)} \\ \end{array} $$

where $j=1, 2, \ldots, J$, *IC*_*j*_ and *RC*_*j*_ denote the irrigated and rainfed crops cultivable in province *j*, respectively.

(ii) Constraint on cultivation area

A fraction of *ICA*_*j*_^*free*^ can be used in irrigated lands:

28 $$ 0 \le M \times \sum\limits_{i = 1}^{51} {{(X}_{1(i,j)} \times ICA_{(i,j)} ) \le ICA_{j}^{free} } \begin{array}{*{20}c} , & {0 \le M \le 1} & {} \\ \end{array} $$

where $j=1, 2, \ldots, J$, and *M* denotes the fraction of blue water available.

(iii) Constraint on water use

The amount of water used to modify the cropping pattern in the provinces is limited:

29 $$ \sum\limits_{i = 1}^{51} {TWF_{blue(i,j)}^{m} \le RWR_{j} - \sum\limits_{i = 1}^{51} {TWF_{blue(i,j)} + } } TWF_{blue,j}^{free} $$

where $\left(j=1,2,3,\ldots,J\right),$ *TWF*^*m*^_*blue(i,j)*_ denotes the blue WFs used to modify the cultivation in province *j*, and *TWF*_*blue(i,j)*_ represents the initial blue WFs consumed in province *j* to cultivate crops before changing the cropping pattern.

### Ideal solution and pareto optimality

This work applied the multi-objective optimization Non-dominated Sorting Genetic Algorithm-II (NSGA-II). The NSGA is based on the Genetic Evolutionary Algorithm and the Selection, Crossover, and Mutation operations^50^. The NSGA was introduced by Deb et al.^51^,Srinivas and Deb^52^, then improved to the NSGA-II^51^. The NSGA-II has been widely studied in water resources management^53–55^.

The NSGA-II produces a Pareto front of solutions, in which, each point represents a management scenario. The decision-maker selects a scenario based on the objective functions and situational analysis. Multi-criteria decision-making methods (MCDM) can be applied to select an efficient point on the Pareto front curve^56,57^. This work implements the technique for order preference by similarity to ideal solution (TOPSIS) as the MCDM employed for that purpose. A description of the TOPSIS method is presented in the appendix.

**Figure 1:**
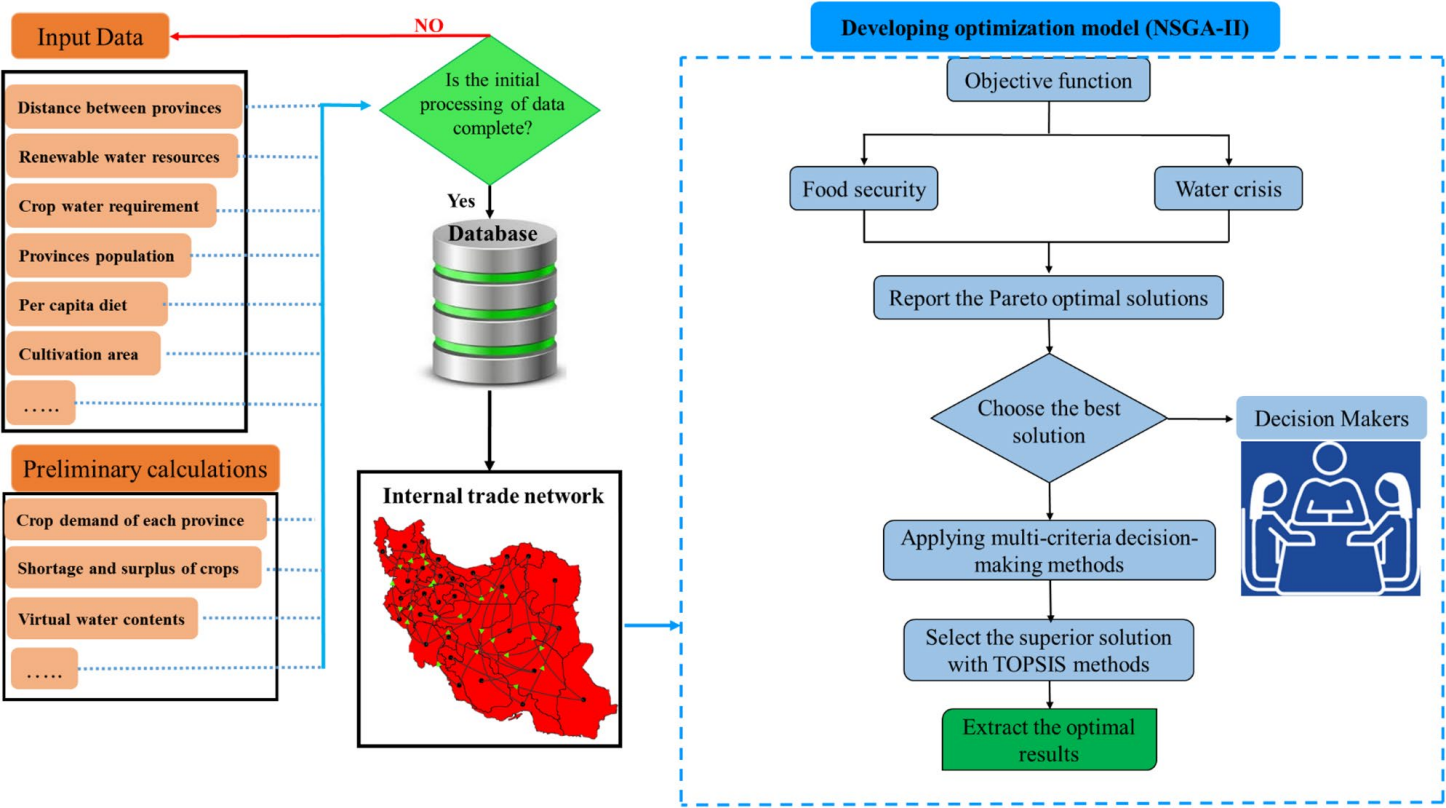
Flowchart of the methodology.

The NSGA-II parameters were determined based on a trial-and-error process. Multiple runs of the algorithm were used to adjust the parameters to reduce uncertainty. For this purpose, the population size and maximum iteration were set equal to 400 and 500, respectively, and the crossover and mutation rates were set equal to 0.8 and 0.1, respectively. The flowchart of the proposed approach is displayed in Fig. 1.

## Study area and data sources

**Figure 2:**
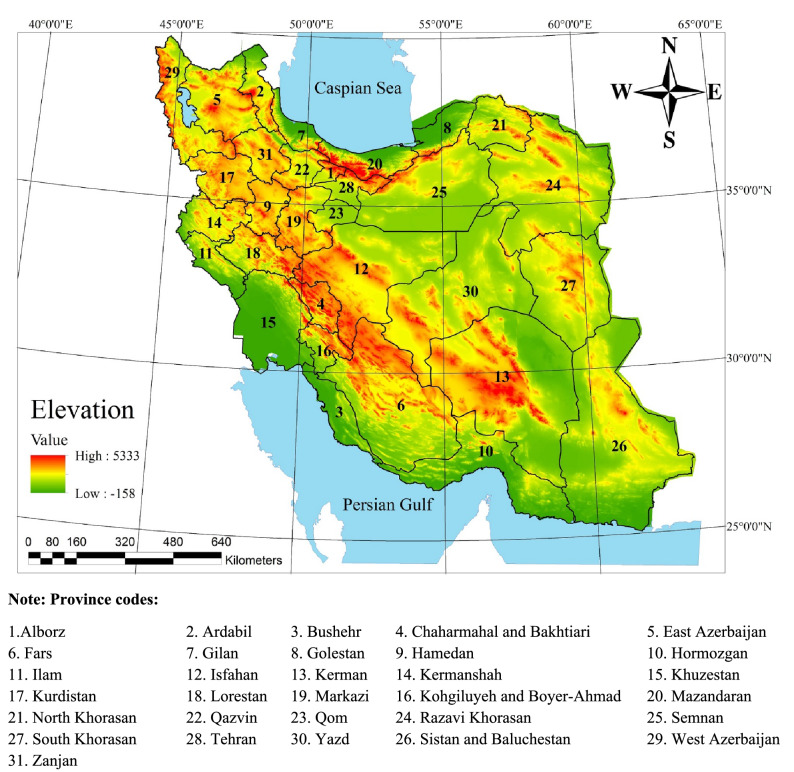
Map of the study area. (ArcGIS 10.3.1).

Iran is located between the latitude of 25° 00′ N and 38° 39′ N and longitude of 44° 00′ E and 63° 25′ with an area of 1,648,000 km^2^, which is divided into 31 provinces (Fig. 2). The average of minimum and maximum temperatures in 2017 were 11.2 °C and 24.5 °C, respectively. The average annual precipitation is 275 mm, with the highest and lowest amounts occurring in the provinces Gilan (522 mm), Sistan and Baluchestan (83 mm), respectively. This work applied data on 51 crops (see supplementary data) including rainfed and irrigated cultivation areas, crop yield, and production rate on a provincial scale, and crop coefficient based on crop type and development stages (initial, middle, and late stages) obtained from the Ministry of Agriculture of Iran for each province^23^. Other information on renewable water resources, meteorological variables, population, and spatial features of the provinces was gathered from the Iran Water Resources Management Company and the Meteorological Organization and Statistics Center of Iran^23,58–60^. The data for 2017 were used to evaluate the performance of the proposed crop optimization method. The proposed approach is time-independent; 2017 was chosen to evaluate the performance of the approach. Selected crops included all agricultural and horticultural crops of Iran, which were categorized in eight groups: (i) cereals; (ii) forage plants; (iii) vegetables; (iv) oil crops; (v) sugar crops; (vi) pharmaceutical crops; (vii) nuts, and (viii) fruits.

## Results and discussion

### Interprovincial crop trade network

Nine crops among all those produced in 2017, i.e., wheat, barley, rice, corn, tea, potatoes, beans, lentils, and soybeans, were exported from and imported into the country. In other words, the export seemed to occur without considering the internal demands. A *WF*_*blue*_ volume of 781 × 10^6^ m^3^ was consumed to produce these crops. This amount of blue water was deemed exported from the country because of the lack of an internal trade network to distribute the crops to meet the demands of the country for those crops. This problem occurs regularly, and this work attempts to remedy this inefficient situation and save *WF*_*blue*_.

**Figure 3:**
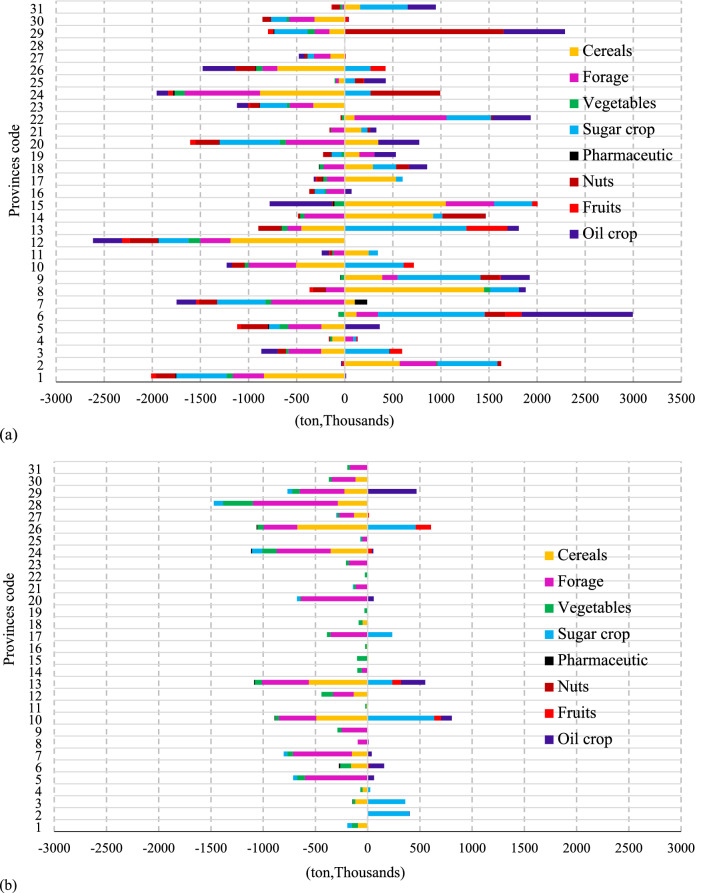
Deficit and surplus of crops without (**a**) and with (**b**) the trade network (ArcGIS 10.3.1**).**

**Figure 4:**
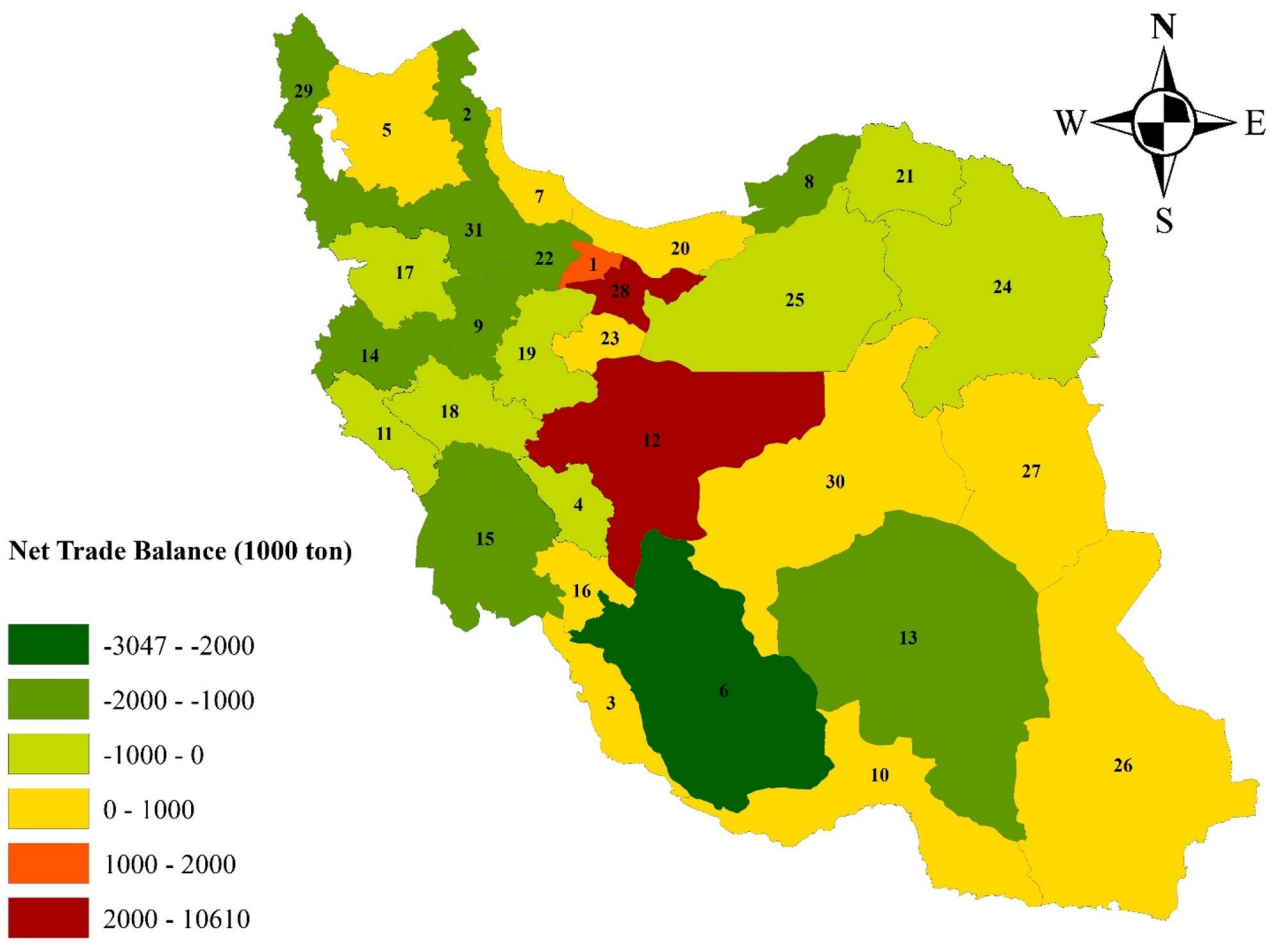
Spatial visualization of net crop trade by province: Positive sign denotes imports; negative sign denotes exports (ArcGIS 10.3.1).

Figure 3 shows the deficit and surplus of crops before and after applying the trade network. It can be seen that the trade network establishes suitable links among the provinces and reduces their deficits. Tehran province is not included in Fig. 3a (Code 28 in the map) because it had a severe deficit of all crops and its value (12.1 × 10^6^ tons) plots outside the chart’s scale. However, it is seen from Fig. 3b that crop deficits in Tehran province were largely eliminated. Figure 4 presents the spatial visualization of the net crop trade by province, where the negative sign denotes provinces that export crops, which mainly include the country’s northeastern and western provinces. Figure 4 shows that the northern provinces, including Gilan (Code 7) and Mazandaran (Code 20), have net imports. This trend is also noted in Qasemipour et al.^61^'s study that the northern provinces that do not have water scarcity have imported products, and the provinces with water scarcity have exported products. Fars (Code 6) and Tehran (Code 28) provinces would have the largest interprovincial exports and imports, respectively. The information concerning the province exports and imports for several crop groups is presented in Table 1. Tehran province would have the largest amounts of imports except for oil products, because there is a large population in this province. Most of the strategic crops that form the basis of food security are cereals. Golestan (Code 8) plays a significant role with respect to cereals as it exports 1.4 × 10^6^ tons of cereals. This table also shows that provinces such as Fars and Kerman, which have a dry climate, have the highest exports of fruits and nuts in Iran, and Karandish and Hoekstra^18^ has already approved this result that these regions are the main exporters of this category of crops despite the lack of water resources.

**Table 1:** Leading exports and imports by crop group.

Group of crops	Maximum export		Maximum import	
	10^3^ ton	Province (code)	10^3^ ton	Province (code)
Cereals	1449.2	Golestan (8)	3758.1	Tehran (28)
Forage plants	957.2	Qazvin (22)	1570.3	Tehran (28)
Vegetables	1110.6	Fars (6)	2646.7	Tehran (28)
Oil crops	63.893	Golestan (8)	51.698	Mazandaran (20)
Sugar crops	1651.435	West Azerbaijan (29)	993.518	Tehran (28)
Pharmaceutical	127.562	Gilan (7)	39.191	Tehran (28)
Nuts	343.666	Kerman (13)	258.890	Tehran (28)
Fruits	1011.1	Fars (6)	1305.3	Tehran (28)

The province codes refer to the provinces shown on the map in Fig. 2.

The basis of exchange between the provinces under the created trade network is the inter-provincial distance, so that shorter distances imply large exchanges that reduce the transportation cost. Faramarzi et al.^40^has also considered organizing an interprovincial trade network for Iran, which has primarily supplied wheat deficit in the provinces.

### Water footprint trade balance

**Figure 5:**
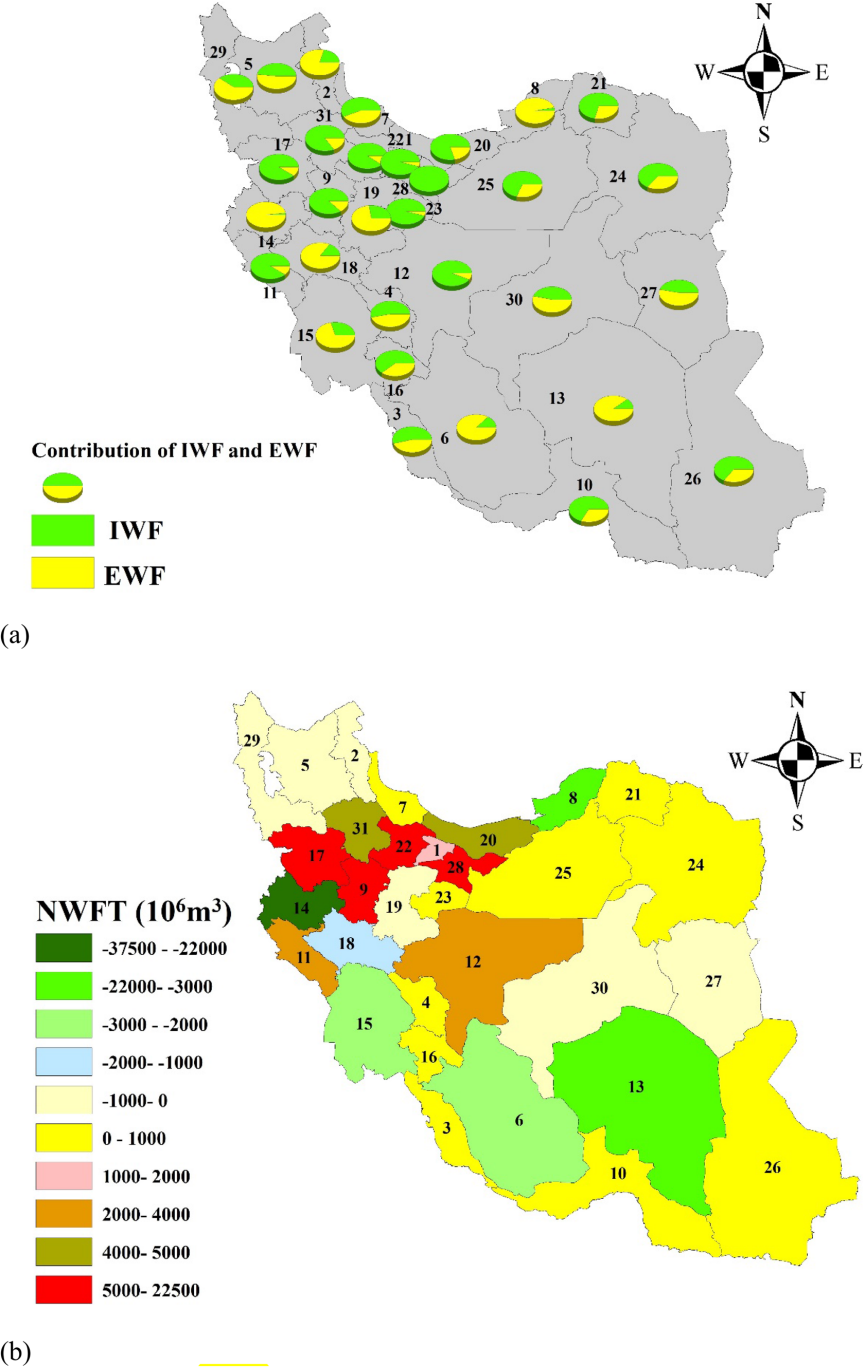
The shares of exported (EWF) and imported (IWF) water footprints (**a**), and net water footprints trade (NWFT) (**b**) Positive sign denotes net water footprint imports; a negative sign denotes net water footprints export (ArcGIS 10.3.1).

The share of total WFs exported (*EWF*) from and imported (*IWF*) to each province, and the net water footprint trade is depicted in Fig. 5. The provinces’ shares in supplying crop demands are also displayed in Fig. 5. Tehran province (Code 28) imports exclusively, while Kermanshah province (Code 14) exports exclusively. The first three ranks of the largest water footprint (WF) exports and imports relative to the amounts of exchanged crops are listed in Table 2. The largest export water footprints (*EWFs*) correspond to Kermanshah, Golestan, and Kerman provinces with 38,155, 22,637, and 4415 × 10^6^ m^3^, respectively; however, most of the exported crops would be from Fars, Hamedan, and Qazvin provinces. Comparison of the import water footprints (*IWFs*) with the imported crop volumes indicates that the province with the most crop exports would not necessarily have the largest *EWF* (except Tehran). This happens because it can exchange crops with lower water footprint per mass, and the same holds for importing provinces. Eventually, the exchange of crops between the provinces would continue for as long as there is a surplus crop for which some province needs it. Surplus crops per province are shown in Fig. 3b. These crops would normally be the country's international exports.

**Table 2:** Main water footprint exports and imports compared with the amounts of exchanged crops.

Maximum IWF		Maximum EWF	
10^6^ m^3^	Province (code)	10^6^ m^3^	Province (code)
22,480.1	Tehran (28)	38,154.7	Kermanshah (14)
11,188.8	Hamedan (9)	22,637.1	Golestan (8)
10,374.6	Kurdistan (17)	4415	Kerman (13)

Maximum import		Maximum export	
10^3^ ton	Province (code)	10^3^ ton	Province (code)
10,608.2	Tehran (28)	3047.1	Fars (6)
2175	Isfahan (12)	2165	Hamedan (9)
1803.1	Alborz (1)	1917.4	Qazvin (22)

**Figure 6:**
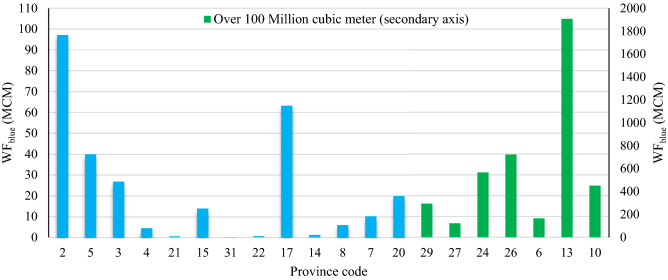
Total water footprints available to modify the cropping pattern by province. *Note* The province codes refer to the provinces shown on the map in Fig. 2.

The new methodology assumes that the international export of crops is modified to improve food security and self-sufficiency, which is a macro-economic policy. This export modification prevents the export of 4510 × 10^6^ m^3^ of *WF*_*blue*_, which is shown in Fig. 6 by province. Exports of crops with a lot of virtual water abroad have also been remarked in Mohammadi-Kanigolzar et al.^62^'s research, which shows mismanagement in Iran.

The water saved by all provinces was in the form of *WF*_*blue*_ (except 1755 m^3^ in Mazandaran province). Twenty provinces apply *WF*_*blue*_ for the cultivation of new crops, among which Kerman with 1907 × 10^6^ m^3^ and Zanjan with 0.1 × 10^6^ m^3^ have the largest and lowest available *WF*_*blue*_, respectively. Modified cropping patterns were identified by the methodology to achieve food security and avert water crises.

### Optimal cropping pattern with proposed modifications

The NSGA-II was applied to modify cropping patterns based on two objectives: improving food security (*FS*) and averting water crises (*WC*). The optimization was repeated 10 times to capture the variability of solutions given the random nature of the NSGA-II. Each optimization run took more than 10 h. The similarity of the Pareto fronts across runs demonstrated the robustness of solutions. The land available to modify the cropping pattern in the provinces was based on their surplus crops [Eqs. (22)–(24)]. Only crops with deficit conditions under the traditional cropping pattern were selected for cultivation.

**Figure 7:**
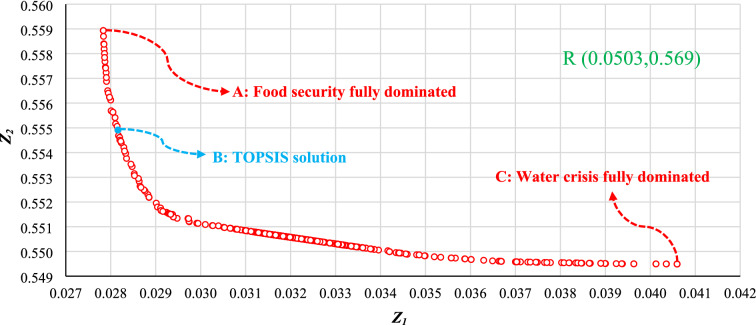
Pareto front solution set.

Figure 7 shows the Pareto front for 400 solutions that are evenly distributed. Each solution represents a management scenario based on two objectives, and points A and C show the optimal cropping patterns based on the objective function *Z*_1_ alone [Eq. (18)] and the function *Z*_2_ alone [Eq. (19)], respectively. Optimal solutions close to point A preferentially minimize *Z*_1_ (i.e., maximizing food security). In contrast, optimal solutions close to point C reduce the water crisis. In Fig. 7, the coordinates for point R of the objective functions for the reference situation (before cropping pattern modifications) are shown, and it is clear that point R is outside the Pareto Front range. Therefore, the Pareto solutions would modify the reference situation. Point A has an improvement of 45% and 2% over R at *Z*_1_ and *Z*_2_, respectively. At point C this improvement is equal to 19% and 3% over R at *Z*_1_ and *Z*_2_, respectively. Despite the small changes in *Z*_2_, the range of *Z*_1_ in Fig. 7 indicates the model effectiveness in selecting the crops with high productivity and yield. The next step is to apply the TOPSIS MCDM to choose the best solution from the Pareto front. Point B on the Pareto front (Fig. 7) was selected based on TOPSIS., It is clear that TOPSIS chose an optimal solution that maximized food security given the position of point B on the Pareto front. The difference in the percentage improvement of functions *Z*_1_ (26%) and *Z*_2_ (1%) between points A and C confirms the TOPSIS result, because by choosing a point closer to point A there would be a greater improvement in *Z*_1_ than the improvement in *Z*_2_ that would be realized by choosing a point closer to C.

### State of water crisis and food security with cropping pattern modification

**Figure 8:**
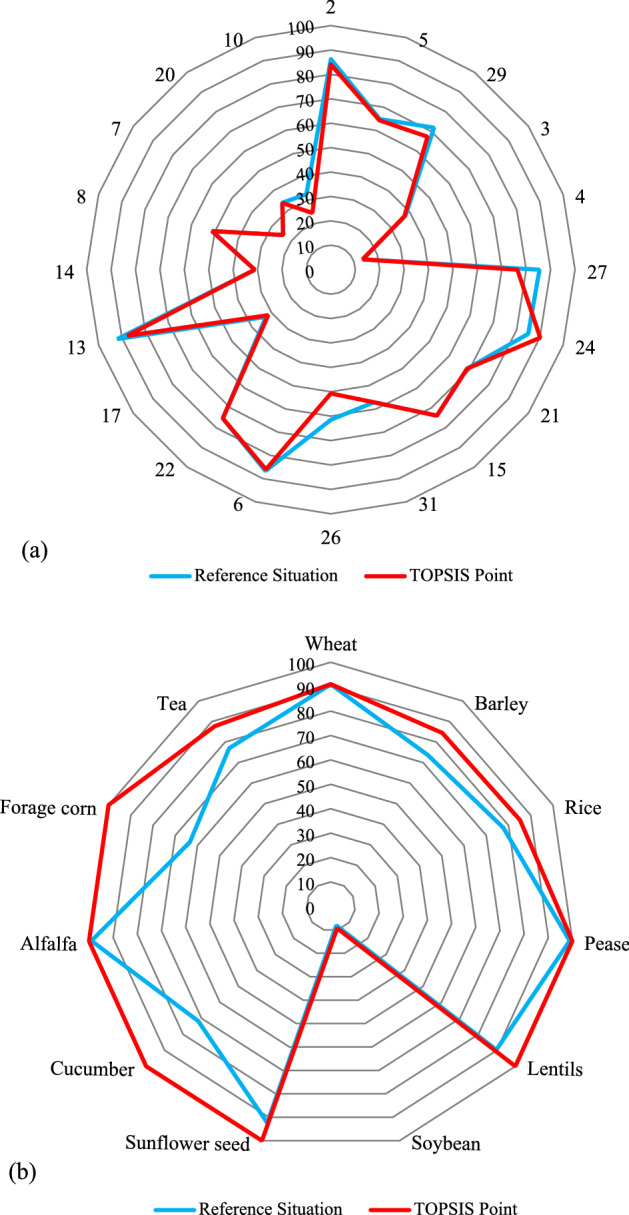
Comparison of water crisis percentage (**a**), and food security percentage (**b**) in the reference and post-modification conditions. *Note* The numbers in Fig. 9a refer to the province codes.

A total of 400 management scenarios were created by the Pareto front so that the decision-maker can choose one of them to reduce country's international exports in order to promote self-sufficiency and food security. This work evaluated point B as a management scenario to be compared with the reference situation. The decision-maker can choose any of the 400 scenarios based on the country's situation and existing policies. Point B was considered here as one of the best scenarios based on the TOPSIS approach. Point B would improve the *FS* and *WC* objectives by 44% and 2.5%, respectively, compared to the reference situation. The produced crops (tons) obtained from the cropping pattern modifications for different crops and provinces corresponding to point B are listed in Table 3. The maximum production corresponded to forage crops with 6,533,776 tons, followed by cereals with 825,724.8 tons. Interestingly, there would be no production in the nuts group and vegetables with 1498.7 tons would have the second-lowest production. Figure 8 compares the reference and post-modification conditions based on the percentage of water crisis [Eq. (17)] and food security of crops with deficit conditions in the country [Eq. (16)]. The effectiveness of the proposed approach is seen in Fig. 8. The increase in the percentage of food security for 10 crops is shown in Fig. 8, other crops would reach 100% supply relying on the country's primary production and the trade network. The self-sufficiency policy in Delpasand et al.^44^ research also provided 50% of the crop demand, which is a low amount compared to the present study.

**Table 3:** Crop production (tons) under a modified cropping pattern.

Code	Province	Cereals	Forage plants	Vegetables	Oil crops	Sugar crops	Pharmaceutical	Nuts	Fruits
2	Ardabil	29,642.8	60.8	0	8653.2	283.7	0	0	0
5	East Azerbaijan	14,370.6	96.1	2.7	2	42.6	0	0	0
29	West Azerbaijan	100,055.5	11,518.7	6.5	0	0	0	0	0
3	Bushehr	24,445.6	322.8	0	0	0	0	0	0
4	Chaharmahal and Bakhtiari	1893.7	381.3	0	0	128.8	0	0	0
27	South Khorasan	2.8	0	0.2	0	0	0	0	8717
24	Razavi Khorasan	231,851.7	1,411,650.9	0	0.5	0.5	0	0	0
21	North Khorasan	120.3	45.6	11.2	1.3	8.3	0	0	0
15	Khuzestan	2913.8	12,774.5	417.7	30.3	28.8	0	0	2.1
31	Zanjan	54.5	24.1	17.8	0	13.9	0	0	0
26	Sistan and Baluchestan	55,251.4	732,671.9	0	0	0	0	0	0
6	Fars	56,683.8	8097.5	149.2	1655.3	2443.2	0	0	0
22	Qazvin	9.4	62.5	20.9	127.3	5.5	0	0	0
17	Kurdistan	22,628.6	236.4	0	0	54.1	0	0	0
13	Kerman	236,539.3	3,945,670.1	0	0	0	0	0	0
14	Kermanshah	697.6	21	0	1.8	46.4	0	0	12
8	Golestan	2188.0	131	83.9	20.8	25.2	0	0	0
7	Gilan	20.7	0.1	1.4	0	0	19,442	0	0
20	Mazandaran	7669.5	230.4	787.2	2171.7	0.3	2.9	0	0
10	Hormozgan	38,684.6	409,780.5	0	0	0	0	0	0

The province codes refer to the provinces shown on the map in Fig. 2.

**Figure 9:**
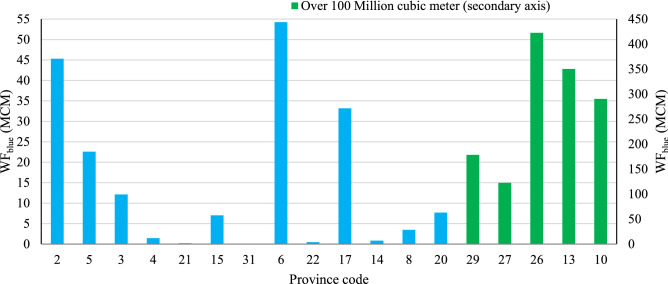
Water footprints saved in the provinces.

**Figure 10:**
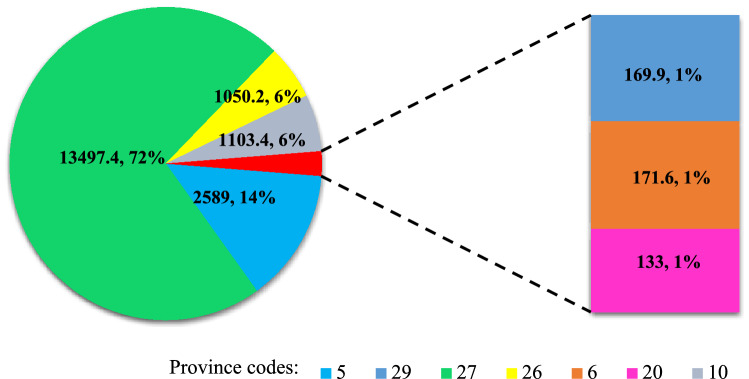
Amount of free land by province. *Note* The numbers on the map refer to the amount of free land (ha) and the share of total free land (%).

Despite the larger production the proposed method would reduce the water crisis in all provinces with surplus crops (18 provinces) except for Razavi Khorasan (Code 24) and Gilan (Code 7) (Fig. 8a). These results indicate improvements in water productivity associated with a modified cropping pattern. The *WF*_*blue*_ needed for production with a modified cropping pattern would be 3303 × 10^6^ m^3^, which compares with 4510 × 10^6^ m^3^ with the reference cropping pattern. Although the total water footprint consumed to produce crops in the country was 60 × 10^9^ m^3^, this consumption was reported by Karandish and Hoekstra^18^ for 2010 as 57 × 10^9^ m^3^. Figure 9 shows the water that would be saved in different provinces. The largest savings would occur in Sistan and Baluchestan provinces with 422 × 10^6^ m^3^, while Zanjan had the lowest savings with 0.02 × 10^6^ m^3^. Among the provinces with surplus *WF*_*blue*_ (Fig. 6), only Razavi Khorasan and Gilan provinces would not save water and would have an increase of 5% and 0.07% in WC, respectively. These provinces would use the surplus *WF*_*blue*_ with a modified cropping pattern and would use the province's renewable water resources according to the water-consumption constraints [Eq. (29)]. It may seem that Point B as a management scenario is similar to Iran's current policy, which prioritizes self-sufficiency and food security. In contrast, one of the objective functions of the proposed method is averting water crises. Under the reference situation, all available land is used to produce crops, regardless of the impact on the water crisis. The modified cropping patterns leave some areas fallow, resulting in a reduction in the cropland extent (Fig. 10). The option of having fallow land allows the decision-maker to cultivate or not cultivate according to changing conditions and the consequences of heightened use of water resources. In the case that the decision-maker leaves fallow land uncultivated the government may provide support to compensate farmers for the loss of income, as was done in agricultural lands of the Urmia Lake basin^63^. The total fallow land equals 19 × 10^3^ ha, which is distributed among 7 provinces. The largest portions of fallow land would be in South Khorasan (Code 27) with 13,497 ha and East Azerbaijan (Code 5) with 2589 ha.

### Results in terms of Iran’s food and water policies

One of the main goals is to achieve food self-sufficiency in Iran by increasing access to water resources and expanding croplands^18^. There has been a rising exploitation of water resources in recent decades and less attention has been paid to management actions and changes to the agricultural system. This study’s results demonstrated that an integrated approach based on inter-provincial trade network and structural modifications in the agricultural system would assure food self-sufficiency and control the water crisis. Cropping pattern modifications and associated increased water productivity and decreasing *WF*_*blue*_ per ton of crops would be effective in controlling water crisis, something that has been reported previously in studies involving other regions [see, e.g., Lopez and Bautista-Capetillo^64^ and Zhuo et al.^65^]. In fact, the weakness of Iranian policy is separating the trade network, food self-sufficiency, and water crisis. This study’s method optimizes water use in agriculture and avoids unnecessary trade flows in Iran, in other countries, and in international trade. Reducing water use applying the proposed approach is achieved by changing the cropping pattern of agricultural lands and balancing the trade network. Therefore, farmers cannot use the saved water to expand the cultivated land because no land is available, as it has already been cultivated. If the land is available (fallow land), the farmer receives government support not to cultivate. Also, water use is monitoring with intelligent volumetric meters, which precludes wasteful use of water.

This study acknowledges that changes in the trade network are not easily achieved in spite of physical and human capital investments; however, the results of the proposed approach provide guidelines for change that would lead to more efficient investment of resources.

## Conclusions

The present study developed a systematic decision-support framework by linking the trade network, food security, and water crisis. The framework is demonstrated with its application in Iran. The results in the first stage involving the creation of a trade network showed the saving of 781 × 10^6^ m^3^ of blue water. This amount of *WF*_*blue*_ is normally unavailable due to inconsistencies in the country's trade network. In the second stage, the results of optimization of the cropping patterns were presented as a Pareto front and compared with the reference situation. The Pareto front consisted of 400 management scenarios for cropping pattern modifications, which imply improvements of 19–45% and 2–3% in food security and water stress levels (blue WFs), respectively. This study introduced a superior scenario that would improve food security by 44% and decrease blue WFs by 2.5%. This could save 1207 × 10^6^ m^3^ of blue water in the country. Iran's macro-policy seeks to achieve food self-sufficiency, and this potentially contradicts the reduction of the water stress. Therefore, water resources have received secondary attention. The proposed method evaluated the self-sufficiency policy while reducing the water crisis, and provided promising results. This paper applied its methodology to Iran's water use and food production, yet, the methodology is widely applicable. For example, most Middle Eastern countries are dealing with similar problems as those faced by Iran, and, therefore might profit from similar solutions. Moreover, the international trade network might profit from this paper’s method by reducing the WFs and virtual water flows.

## Limitations of the study and further research

Despite valuable results from water footprint research, there remain limitations. This research is no exception to these limitations. The main limitations are data availability, quality and accuracy, and the set of assumptions used in the algorithm and methodology. It is assumed when calculating the water footprint components that all the irrigation water requirements are fully provided in irrigated cultivation. Also, gray water footprint was not considered due to the unavailability of data. Uncertainty influences the results, and it affects input data, algorithm parameters, the initial population, and the number of iterations of the algorithm. Sensitivity and uncertainty analyses may be applied to evaluate the effect of uncertainty, but they are techniques that require substantial computational and time investments. This work attempted to reduce the effect of uncertainty with multiple runs and by extracting reliable results from the algorithm. Climate change and its impact on water resources and crop yields in irrigated and rainfed cultivation was not investigated in this study. However, future research could cover all of these and other issues, including industrial products, aquaculture, livestock, and related goods and services. Each of the limitations could be addressed in a separate study.

A few points are worth considering regarding applying the current approach in other regions with similar conditions to those prevailing in Iran. Researchers could prepare information based on the flowchart of the present study (Fig. 1) and reduce the cited limitations to the extent possible. By following this work’s approach one can to obtain results for other regions with updated data. The domestic trade network in the region would be created with the additional information on demand and production. The results of the trade network are input to optimization algorithm. Lastly, the optimal cropping pattern of any region is determined according to the scenarios of food security and water crisis. Thus, analysts can create multiple management scenarios (Pareto front) for decision-makers. The advantage of this work’s approach is that it is independent of input information and, therefore can be easily employed by other researchers in other regions.

## Supplementary Information

Supplementary Information 1.

Supplementary Information 2.

Supplementary Information 3.
