# Supplementary material for: Optimal virtual water flows for improved food security in water-scarce countries
Source: Sci Rep. 2021 Oct 25;11:21027. doi: 10.1038/s41598-021-00500-6 (PMC8546057; doi:10.1038/s41598-021-00500-6)
Supplement: Supplementary file 1 — Supplementary Information 1. [file 41598_2021_500_MOESM1_ESM.docx]

**Appendix**

The use of TOPSIS in this study is due to the advantages of MCDM methods. One of these advantages their dynamic nature, which can be improved over time thus providing capacity to adapt to new conditions. The TOPSIS method seeks to determine the positive (I^+^) and the negative (I^-^) ideal solutions and based on them, this method identifies the best solution that has the closest and farthest distances from I^+^ and I^-^, respectively. The steps of the TOPSIS method are as follows (Hwang and Yoon, 1981):

1. Formation of solution matrix: Each point of Pareto front curve is a solution that has a value for the first and second objective functions.
2. Normalizing the solution matrix: The equation for normalizing the solutions is as follows:

| *(A.1)* | $X_{(m\times n)}^{norm}=\frac{X_{(m\times n)}}{\sqrt{\sum_{m=1}^{m} X_{(m\times n)}^{2}}}\begin{matrix} & (m=1,2,3,...,m;\begin{matrix} n=1,2) & \end{matrix} \end{matrix}$ |
| --- | --- |

where, *X_(n × m)_* denotes the solution in the *m^th^* row and the *n^th^* objective function (column) and *X^norm^_(m×n)_* denotes the normalized solution.

1. Determining the weight of objective functions: the TOPSIS method calculates the best solution in the Pareto front curve based on the distance from the ideal solutions and the weight of the objective functions. TOPSIS does not set the weights for theobjective functions, which is done by the decision-maker. This work applies the Shannon Entropy method (Shannon, 1948) to weight the objective functions. Entropy theory assumes that the occurrence probability is inversely related to the amount of information. The entropy is a statistical index of randomness or uncertainty [See Maroufpoor et al., (2020) for more information]. The function with more entropy has less weight. The weighted solutions [*W_(m × n)_*]. are created by multiplying the weights by *X^norm^_(m×n)._*
2. Calculation of the distance of solutions from *I^+^* and *I^-^*: For minimum objective functions, *I^+^* and *I^-^* will be the minimum and maximum values ​​of the function, respectively. The distance from *I^+^* and *I^-^* is determined with the following equations:

| *(A.2)* | $D_{m}^{+}=\begin{matrix} \sqrt{\sum_{n=1}^{2} \left(W_{(m\times n)}-I_{n}^{+}\right)^{2}} & (m=1,2,3,...,m;\begin{matrix} n=1,2) & \end{matrix} \end{matrix}$ |
| --- | --- |
| *(A.3)* | $D_{m}^{-}=\begin{matrix} \sqrt{\sum_{n=1}^{2} \left(W_{(m\times n)}-I_{n}^{-}\right)^{2}} & (m=1,2,3,...,m;\begin{matrix} n=1,2) & \end{matrix} \end{matrix}$ |

where, *D^+^_m_* and *D^-^_m_* denote the distances of the *m^th^* solution in *n^th^* objective function from *I^+^* and *I^-^*, respectively. Finally, the relative closeness of each solution on the Pareto front curve is calculated as follows:

| *(A.4)* | $R_{m}=\frac{D_{m}^{-}}{D_{m}^{-}+D_{m}^{+}}$ |
| --- | --- |

where *R_m_* denotes the relative closeness of the *m^th^* solution. The relative closeness range is 1˂*R_m_*˂0, and the best solution is the closest to 1.
